# Dysfunctional variants of *ABCG2* create strong individual and population risks for progression of hyperuricemia: the potential for implementation of genome-personalized nursing

**DOI:** 10.1007/s13577-025-01310-y

**Published:** 2025-12-26

**Authors:** Akiyoshi Nakayama, Kimiko Hayano, Miki Ueno, Yuka Miyoshi, Hiroshi Nakashima, Seiko Shimizu, Itsumi Hashimoto, Miho Toba, Rion Kikuchi, Nana Takehana, Michiru Miura, Yusuke Kawamura, Yu Toyoda, Tomoko Mizuno, Risa Tanabe, Yoshinobu Hamada, Takashi Tamura, Yasufumi Kato, Yoko Mitsuda, Hirofumi Nakaoka, Ken Yamamoto, Masashi Tsunoda, Nariyoshi Shinomiya, Hirotaka Matsuo

**Affiliations:** 1 Department of Integrative Physiology and Bio-Nano Medicine, National Defense Medical College, 3-2 Namiki, Tokorozawa, Saitama 359-8513 Japan; 2 International Research Collaboration Officer, National Defense Medical College Research Institute, National Defense Medical College, Tokorozawa, Japan; 3 Department of Community Health Nursing, National Defense Medical College, Tokorozawa, Japan; 4 Department of Nursing, National Defense Medical College, Tokorozawa, Japan; 5 Department of Preventive Medicine and Public Health, National Defense Medical College, Tokorozawa, Japan; 6 Research Division, Maritime Self-Defense Force Undersea Medical Center, Yokosuka, Japan; 7 Department of Obstetrics and Gynecology, National Defense Medical College, Tokorozawa, Japan; 8 Department of Preventive Medicine, Nagoya University Graduate School of Medicine, Nagoya, Japan; 9 Department of Cancer Genome Research, Sasaki Institute, Sasaki Foundation, Tokyo, Japan; 10 Department of Biomedical Data Science, Kagoshima University Graduate School of Medical and Dental Sciences, Kagoshima, Japan; 11 Department of Medical Biochemistry, Kurume University School of Medicine, Kurume, Fukuoka Japan; 12 Department of Biomedical Information Management, National Defense Medical College Research Institute, National Defense Medical College, Tokorozawa, Japan

**Keywords:** Hyperuricemia/gout, Population-attributable fraction/population-attributable risk percent (PAF/PAR%), Genome-personalized nursing, Health-behavior intervention model, Cognitive–behavioral theory (CBT)

## Abstract

Gout, a common disease, develops after prolonged hyperuricemia with elevated serum uric acid (SUA) levels. Sex, overweight/obesity, heavy drinking, and aging all increase risk. Common dysfunctional variants (polymorphisms) in the *ABCG2/BCRP* gene are major genetic causes of gout/hyperuricemia. Early management would thus have potential benefits for public health. Several studies report the effectiveness of nurse-led care for gout management. We evaluate here the individual and population genetic effects of *ABCG2* in 9244 Japanese study participants, in all of whom *ABCG2* variants had a higher population-attributable fraction (PAF; approximately 30%) for progression of hyperuricemia than those for other typical environmental risk factors (overweight/obesity, heavy drinking, and aging). PAFs for aging in males and heavy drinking in females were not significant. All these factors also showed significant individual risk of increased SUA level, and *ABCG2* variants had sufficient effect size to allow them to be converted into other environmental factors. To implement genome-personalized nursing, we then mapped onto the process a theoretical framework that is based on a psychological model from behavioral change theory. Because the present results for convertibility will improve the predictability of genetic effects, cognitive interventions by nurses based on individual variants could therefore encourage both inducements of behavioral change: efficacy expectations (self-efficacy) and outcome expectations. We conclude that the framework would function effectively based on cognitive interventions by nurses/paramedics. This theoretical framework has potential as a basis for implementing genome-personalized prevention, medicine and nursing of gout/hyperuricemia, and for optimizing nurse-led care.

**Supplementary Information:**

The online version contains supplementary material available at 10.1007/s13577-025-01310-y.

## Introduction

Gout is a common disease characterized by acute arthritis and is caused after prolonged hyperuricemia, determined by elevated serum uric acid (SUA) levels [1]. In addition to the several risk factors for gout reported by Hippocrates 2500 years ago [2], i.e., sex, obesity, alcohol consumption, and aging, we and other researchers revealed that common dysfunctional variants of *ABCG2/BCRP* gene, encoding a high-capacity urate exporter, are significant genetic causes of gout and hyperuricemia [3–5]. We have also reported [6] that two common dysfunctional variants of *ABCG2*, non-functional p.Q126X (rs72552713) and half-functional p.Q141K (rs2231142), create a greater population risk for progression of hyperuricemia than typical environmental risk factors by using population-attributable fraction (PAF, also known as population-attributable risk percent: PAR%). We also evaluated the effect size on SUA by genetic risk by comparing it with environmental factors. Its higher genetic population risk than environmental risk therefore indicates a possible benefit for public health. Our previous study [6], however, did not evaluate effect as divided into each sex, whereas sex appears to be the strongest factor affecting SUA.

Treat-to-target (T2T) care for gout management focuses on maintaining patients’ SUA at below 6 mg/dl [7]. Several studies report that nurse-led T2T care for gout [7–10] achieves improved compliance. This type of care was, however, performed only for patients after gout flares, without the patients’ genetic background being taken into consideration. Due to the increasing global burden of gout [11], more proactive intervention in health behavior needs to be provided, taking account of its genetic factors, to achieve primary prevention. In this study, involving more than 9000 participants, we evaluated the individual and population genetic effects caused by *ABCG2* variants categorized by sex.

We also mapped a theoretical framework onto Bandura’s model to be able to implement the results as optimized genome-personalized nursing with the aim of enabling genome-personalized prevention/treatment of the common disease of gout/hyperuricemia. Genetic information has several specific characteristics [12], such as its being “unchangeable,” “predictable,” and “sharable,” which might act as disincentives to good health behavior after the implementation of genome-personalized nursing. “Unchangeable” means that genetic variants do not change over one’s lifetime; “predictable” indicates that the probability of disease onset can be predicted completely or to some extent, dependent on the penetrance of the causative variants. “Sharable” means that variants might be shared among families (biological relatives) and/or ethnic groups. We therefore examined the theoretical framework from the results of the present study and evaluated whether awareness of genetic factors could promote individuals’ appropriate health behavior in response to knowledge of these genetic information characteristics.

## Methods

### Study participants

All the Japanese participants involved in this study were recruited from the Shizuoka and Daiko areas in the Japan Multi-Institutional Collaborative Cohort Study (J-MICC Study; Supplementary Table S1) [13–16]. In all the participants, hyperuricemia was defined as SUA exceeding 7.0 mg/dl (= 420 µmol/l). Several studies [17–19] report that the female population is more vulnerable to increased SUA in terms of progression of metabolic syndrome, so we set the cutoff line for females at 6.0 mg/dl (= 360 µmol/l) if indicated. Alcohol consumption was calculated from the participants’ answers to a written questionnaire, shown in Supplementary Table S2, and those who drank more than 196 g/week (males) and 98 g/week (females) of pure alcohol [20, 21], respectively, were categorized as “heavy drinking.” “Aging” and “overweight/obesity” were defined for those who were ≥ 60 years old and whose BMI was ≥ 25.0 kg/m^2^.

### Genetic analysis

Genomic DNA was extracted from whole peripheral blood cells [22]. Genotyping of the two common variants in the *ABCG2* gene, p.Q126X (c.376C>T, rs72552713) and p.Q141K (c.421C>A, rs2231142), was performed using a TaqMan assay (ThermoFisher Scientific, Waltham, MA, USA) employing a LightCycler 480 (Roche Diagnostics, Mannheim, Germany) as reported in our previous study [23]. Custom TaqMan assay probes were designed as follows: for p.Q126X, VIC-CCACTAATACTTACTTGTACCAC and FAM-CCACTAATACTTACTTATACCAC; for p.Q141K, VIC-CTGCTGAGAACTGTAAGTT and FAM-CTGCTGAGAACTTTAAGTT. The call rates for both variants were 100%, and their minor allele frequencies were 0.0217 and 0.298, respectively. Both variants were in Hardy–Weinberg equilibrium (P > 0.05). Because of no simultaneous presence of the minor alleles of both variants in one haplotype [3, 6], three haplotypes: *1 (Q126 and Q141, haplotype with full function), *2 (Q126 and K141, haplotype with halved function), and *3 (X126 and Q141, non-functioning haplotype) were defined in this study as previously reported [24]. According to the degree of ABCG2 function based on these haplotypes, all the patients could be divided into the following groups: full function (*1/*1), 3/4 function (mild dysfunction; *1/*2), 1/2 function (moderate dysfunction; *1/*3 or *2/*2), and ≤ 1/4 function (severe dysfunction; *2/*3 or *3/*3) [3, 6, 24, 25], as shown in Supplementary Table S3.

### Statistical analyses

R software (version 4.1.0) was used to evaluate the 95% confidence interval (CI) of PAF, and SAS software (version 9.4; SAS Institute, NC, USA) was used for all the other statistical analysis calculations. Linear regression analysis and the Cochran–Armitage test were performed for the association analyses. The P value of the Hardy–Weinberg equilibrium was calculated using the Chi-squared test with Yates’ correction. The PAF for ABCG2 dysfunction and other typical risk factors for hyperuricemia was calculated from the following equation [6]:

$${\text{PAF }} = \, \left[ {\left\{ {\left( {{\text{N}}_{{{\text{HUA}},{\text{Risk}}}} /{\text{N}}_{{{\text{Risk}}}} - {\text{ N}}_{{{\text{HUA}},{\text{NonRisk}}}} /{\text{N}}_{{{\text{NonRisk}}}} } \right) \times \left( {{\text{N}}_{{{\text{Risk}}}} /{\text{N}}_{{{\text{All}}}} } \right)} \right\}/\left( {{\text{N}}_{{{\text{HUA}}}} /{\text{N}}_{{{\text{All}}}} } \right)} \right] \, \times {1}00$$

(“N_HUA,Risk_” and “N_HUA,NonRisk_”, respectively, indicate the numbers of hyperuricemic patients in the risk group and non-risk group. “N_Risk_” and “N_NonRisk_”, respectively, represent the numbers of individuals in the risk group and non-risk group. “N_HUA_” and “N_All_”, respectively, mean the number of all hyperuricemia patients and all participants).

Random resampling methods using computer simulations are often applied to promote statistical robustness [26, 27]. In this study, to evaluate the 95% confidence interval (CI) of the PAF, the bootstrap method [27] was used for random resampling of all participants’ data set, with 10,000 replacements. The linear regression analysis was conducted using a model in which ABCG2 dysfunction, sex, age, BMI, and alcohol consumption were included. We set the threshold for significance to α = 0.05.

### Theoretical framework

For the health-behavior intervention model used in the present study, we adopted a theoretical framework based on a psychological model from a behavioral change theory called “social–cognitive theory (SCT)” formulated by Bandura [28]. The SCT model is a common health education model that is used mainly by public health nurses and is designed to enhance the possibility of behavioral changes that will improve individuals’ health status. The central concept in SCT is “efficacy expectations” (also known as “self-efficacy”), i.e., the conviction that one can successfully execute a behavior that could be arrived at independently based on personal experience. The other concept is “outcome expectations,” which indicates a person’s belief that a given behavior will lead to certain outcomes. SCT regards these two expectations as important controllable inducements to cognitive and behavioral changes, because one’s emotions/mood resulting from cognitive appraisals and self-efficacy leads to behaviors which result in outcomes that match outcome expectations, creating a positive feedback loop [28].

## Results

### High population risk of *ABCG2* variants for progression of hyperuricemia

The present study recruited 9244 Japanese individuals (see Supplementary Table S1 for their characteristics), of whom 1142 (1097 males and 45 females) were hyperuricemic (SUA > 7.0 mg/dl). The number of female patients rose to 211 individuals when hyperuricemia was newly defined as having an SUA of > 6.0 mg/dl. More than a half of the total population of participants (53.3%) had one or two dysfunctional alleles, without obvious differences between the sexes (Supplementary Table S3).

The PAFs of each risk, which indicate the percentage of hyperuricemic patients originating from each risk in the population, were then calculated for participating males, females, and for the whole population (Table 1, Supplementary Fig. S1 and Supplementary Table S4). As was shown in our previous study [6], dysfunctional variants of *ABCG2* create a much higher risk of progression of hyperuricemia [PAF = 30.1%, 95% CI, 24.6–35.6; risk ratio (RR) = 1.81 (95% CI, 1.61–2.03; *P* = 2.85 × 10^–24^)] than other typical environmental risk factors, i.e., overweight/obesity [PAF = 21.7%, 95% CI, 18.5–24.9, RR = 2.48 (95% CI, 2.23–2.77; *P* = 4.52 × 10^–60^)], heavy drinking [PAF = 18.8%, 95% CI, 15.1–22.4, RR = 1.85 (95% CI, 1.65–2.07; *P* = 2.97 × 10^–27^)], and aging [PAF = 3.67%, 95% CI, 0.301–7.04, RR = 1.14 (95% CI, 1.01–1.29; *P* = 0.0281)], although sex difference (male) has the strongest effect [PAF = 91.8%, 95% CI, 89.4–94.1, RR = 22.8 (95% CI, 17.0–30.6; *P* = 2.07 × 10^–225^)]. Similar results were obtained from all participants when hyperuricemia was defined as SUA of > 6.0 mg/dl in females: *ABCG2* variants [PAF = 30.3%, 95% CI, 25.1–35.4, RR = 1.81 (95% CI, 1.63–2.02; *P* = 1.86 × 10^–28^)] had higher impacts than other typical environmental factors (Table 1 and Supplementary Fig. S1).

**Table 1:** PAF on the progression of hyperuricemia for each risk and each sex

Population^a^	Risk factors^b^	PAF (%)	95% CI	Risk ratio	95% CI	*P* value
All (*n* = 9244)	***ABCG2* variants**	30.1	24.6–35.6	1.81	1.61–2.03	2.85 × 10^−24^
	**Overweight/obesity**	21.7	18.5–24.9	2.48	2.23–2.77	4.52 × 10^−60^
	**Heavy drinking**	18.8	15.1–22.4	1.85	1.65–2.07	2.97 × 10^−27^
	**Aging**	3.67	0.301–7.04	1.14	1.01–1.29	0.0281
	**Sex**	91.8	89.4–94.1	22.8	17.0–30.6	2.07 × 10^−225^
All (*n* = 9244) (HUA: SUA > 6 mg/dl in female)	***ABCG2* variants**	30.3	25.1–35.4	1.81	1.63–2.02	1.86 × 10^−28^
	**Overweight/obesity**	20.4	17.4–23.3	2.37	2.14–2.62	9.57 × 10^−62^
	**Heavy drinking**	14.3	11.0–17.7	1.61	1.45–1.79	4.37 × 10^−19^
	**Aging**	6.62	3.49–9.87	1.27	1.14–1.41	1.82 × 10^−5^
	**Sex**	66.6	62.6–70.6	4.86	4.22–5.60	1.97 × 10^−139^
Male (*n* = 4778)	***ABCG2* variants**	30.2	24.9–35.3	1.81	1.62–2.03	9.72 × 10^−27^
	**Overweight/obesity**	14.9	11.6–18.2	1.69	1.52–1.87	6.92 × 10^−22^
	**Heavy drinking**	10.4	6.29–14.1	1.34	1.20–1.49	1.22 × 10^−7^
	Aging	1.89	−1.28–5.07	1.07	0.96–1.20	0.241
Female (*n* = 4466)	***ABCG2* variants**	33.1	2.91–62.5	1.93	1.03–3.61	0.0372
	**Overweight/obesity**	22.2	7.77–37.7	3.49	1.87–6.52	3.11 × 10^−5^
	Heavy drinking	9.14	−3.54–30.0	1.52	0.76–3.01	0.232
	**Aging**	33.8	13.8–53.8	2.95	1.65–5.26	1.33 × 10^−4^
Female (*n* = 4466) (HUA: SUA > 6 mg/dl)	***ABCG2* variants**	31.8	18.5–45.0	1.87	1.41–2.48	1.04 × 10^−5^
	**Overweight/obesity**	19.2	12.6–25.9	3.07	2.31–4.09	1.15 × 10^−14^
	Heavy drinking	20131.65	−8.11–5.07	0.92	0.65–1.30	0.623
	**Aging**	28.7	19.7–37.9	2.54	1.95–3.30	7.32 × 10^−13^

^a^Hyperuricemia (HUA) was defined as having a serum uric acid (SUA) level of over 7 mg/dl for both sexes, and over 6 mg/dl for female if indicated

^b^Each risk factor was set as follows: *ABCG2* variants, having dysfunctional variants of *ABCG2* (X126 and/or K141); aging, ≥ 60 years old; heavy drinking, alcohol consumption > 196 g/week (male) and 98 g/week (female) of pure alcohol; overweight/obesity, BMI ≥ 25.0 kg/m^2^; sex, male. Significant factors are shown in bold

*PAF* population-attributable fraction, *CI* confidence interval

Because sex has the obviously greatest impact, subsequent calculations were performed while categorizing by sex (Table 1, Supplementary Fig. S1 and Supplementary Table S4). In males, PAFs of *ABCG2* variants still had a greater impact on progression of hyperuricemia [PAF = 30.2%, 95% CI, 24.9–35.3, RR = 1.81 (95% CI, 1.62–2.03; *P* = 9.72 × 10^–27^)] than overweight/obesity [PAF = 14.9%, 95% CI, 11.6–18.2, RR = 1.69 (95% CI, 1.52–1.87; *P* = 6.92 × 10^–22^)] or heavy drinking [PAF = 10.4%, 95% CI, 6.29–14.1, RR = 1.34 (95% CI, 1.20–1.49; *P* = 1.22 × 10^–7^)], although aging showed no significant RR (*P* = 0.241). In females, *ABCG2* variants [PAF = 33.1%, 95% CI, 2.91–62.5, RR = 1.93 (95% CI, 1.03–3.61; *P* = 0.0372)], overweight/obesity [PAF = 22.2%, 95% CI, 7.77–37.7, RR = 3.49 (95% CI, 1.87–6.52; *P* = 3.11 × 10^–5^)], and aging [PAF = 33.8%, 95% CI, 13.8–53.8, RR = 2.95 (95% CI, 1.65–5.26; *P* = 1.33 × 10^–4^)] had a significant effect, while heavy drinking did not (*P* = 0.232).

When hyperuricemia was defined as an SUA of > 6.0 mg/dl in females, *ABCG2* variants also showed a significant and stronger effect [PAF = 31.8%, 95% CI, 18.5–45.0, RR = 1.87 (95% CI, 1.41–2.48; *P* = 1.04 × 10^–5^)] than overweight/obesity [PAF = 19.2%, 95% CI, 12.6–25.9, RR = 3.07 (95% CI, 2.31–4.09; *P* = 1.15 × 10^–14^)] and aging [PAF = 28.7%, 95% CI, 19.7–37.9, RR = 2.54 (95% CI, 1.95–3.30; *P* = 7.32 × 10^–13^)]. Heavy drinking still showed no significance in females.

### Effect size of each risk of SUA

To evaluate individual risk, the effect size of SUA levels due to *ABCG2* variants and other risk factors were examined by multiple regression analysis. Among all 9244 participants, 9039 individuals, who received no urate-lowering therapy to treat gout/hyperuricemia, no female hormone replacement therapy, and had no past history of gout, were then recruited. The participants’ SUA rose in both sexes as their ABCG2 function fell (Supplementary Table S3). Regression analyses revealed that all covariates (*ABCG2* variants, BMI, alcohol consumption, age and sex) significantly and independently affected SUA (Table 2 and Supplementary Fig. S2). The effect size of SUA, i.e., regression coefficient (β) due to a 25% decrease in ABCG2 dysfunction, was a gain of 0.180 mg/dl, whereas it was 0.0921 mg/dl per point of BMI, 5.18 × 10^−4^ mg/dl per gram per week of pure ethanol consumption equivalent, 8.75 × 10^−3^ mg/dl per increasing year of age, and 1.44 mg/dl between sexes. The ratio of regression coefficients (β_ABCG2_/β: effect size on SUA due to a 25% decrease in ABCG2 dysfunction vs. each risk factor) showed stronger effects of *ABCG2* variants in terms of ability to raise SUA: a 25% decrease in ABCG2 function had the equivalent effect of an increase in BMI by 1.95 points, alcohol consumption of 347.6 g/week as pure ethanol, or 20.6 years of aging.

**Table 2:** Effect of *ABCG2* variants and other risk factors on SUA levels

Population	Risk factor^a^	β^b^ (regression coefficient)	95% CI	*P* value	β_ABCG2_/β (ratio of regression coefficient)
All (*n* = 9039)	***ABCG2* function**	0.180	0.150–0.210	< 0.0001	1
	**BMI (kg/m**^**2**^**)**	0.0921	0.0847–0.100	< 0.0001	1.95
	**Alcohol consumption (g/week of pure alcohol)**	5.18 × 10^−4^	3.98 × 10^−4^–6.37 × 10^–4^	< 0.0001	347.6
	**Age (years)**	8.75 × 10^−3^	6.42 × 10^−3^–0.0111	< 0.0001	20.6
	**Sex (male)**	1.44	1.40–1.49	< 0.0001	0.125
Male (*n* = 4575)	***ABCG2* function**	0.212	0.164–0.259	< 0.0001	1
	**BMI (kg/m**^**2**^**)**	0.0914	0.0793–0.103	< 0.0001	2.32
	**Alcohol consumption (g/week of pure alcohol)**	4.85 × 10^−4^	3.37 × 10^−4^–6.33 × 10^−4^	< 0.0001	436.4
	**Age (years)**	−5.08 × 10^−3^	−8.84 × 10^−3^–−1.32 × 10^−3^	0.0083	−41.7
Female (*n* = 4464)	***ABCG2* function**	0.147	0.111–0.183	< 0.0001	1
	**BMI (kg/m**^**2**^**)**	0.0861	0.0773–0.0949	< 0.0001	1.71
	**Alcohol consumption (g/week of pure alcohol)**	8.71 × 10^−4^	6.37 × 10^−4^–1.11 × 10^−3^	< 0.0001	169.1
	**Age (years)**	0.0220	0.0193–0.0248	< 0.0001	6.68

^a^Calculation for ABCG2 function was conducted for full function as 1, 3/4 function (mild dysfunction) as 2, 1/2 function (moderate dysfunction) as 3, and ≤ 1/4 function (severe dysfunction) as 4. Calculation for sex was performed for female as 1 and male as 2. Significant factors are shown in bold

^b^“β” indicates the increase of SUA (mg/dl) per unit of each risk factor. The ratio of regression coefficients (β_ABCG2_/β) was calculated from β of ABCG2 function divided by that of each risk factor, showing an effect equivalent to a 25% decrease in ABCG2 function in terms of ability to increase SUA levels

*SUA* serum uric acid level, *BMI* body mass index (calculated as weight in kilograms divided by height in meters squared)

Even after stratifying by sex (Table 2 and Supplementary Fig. S2), all covariates were shown to have significant and independent effects on SUA. In contrast to the results for both sexes, age in males inversely affected SUA, with weak effect size (β = −5.08 × 10^−3^ mg/dl by 1 year of aging) and age in females had a relatively larger effect size on SUA (β = 0.0220 mg/dl by 1 year of aging) than males. Nevertheless, ABCG2 dysfunction still displayed a greater effect than the other environmental risk factors in terms of ability to raise SUA; and a 25% decrease in ABCG2 function had the equivalent effect to a 2.32-point increase in BMI in males or 1.71 points in females, and alcohol consumption of 436.4 g/week in males or 169.1 g/week in females of pure ethanol (Table 2 and Supplementary Fig. S2). These results indicate that genetic factors caused by *ABCG2* variants that affect SUA levels exert greater effects in males than in females.

### Theoretical framework for genome-personalized nursing

**Fig. 1:**
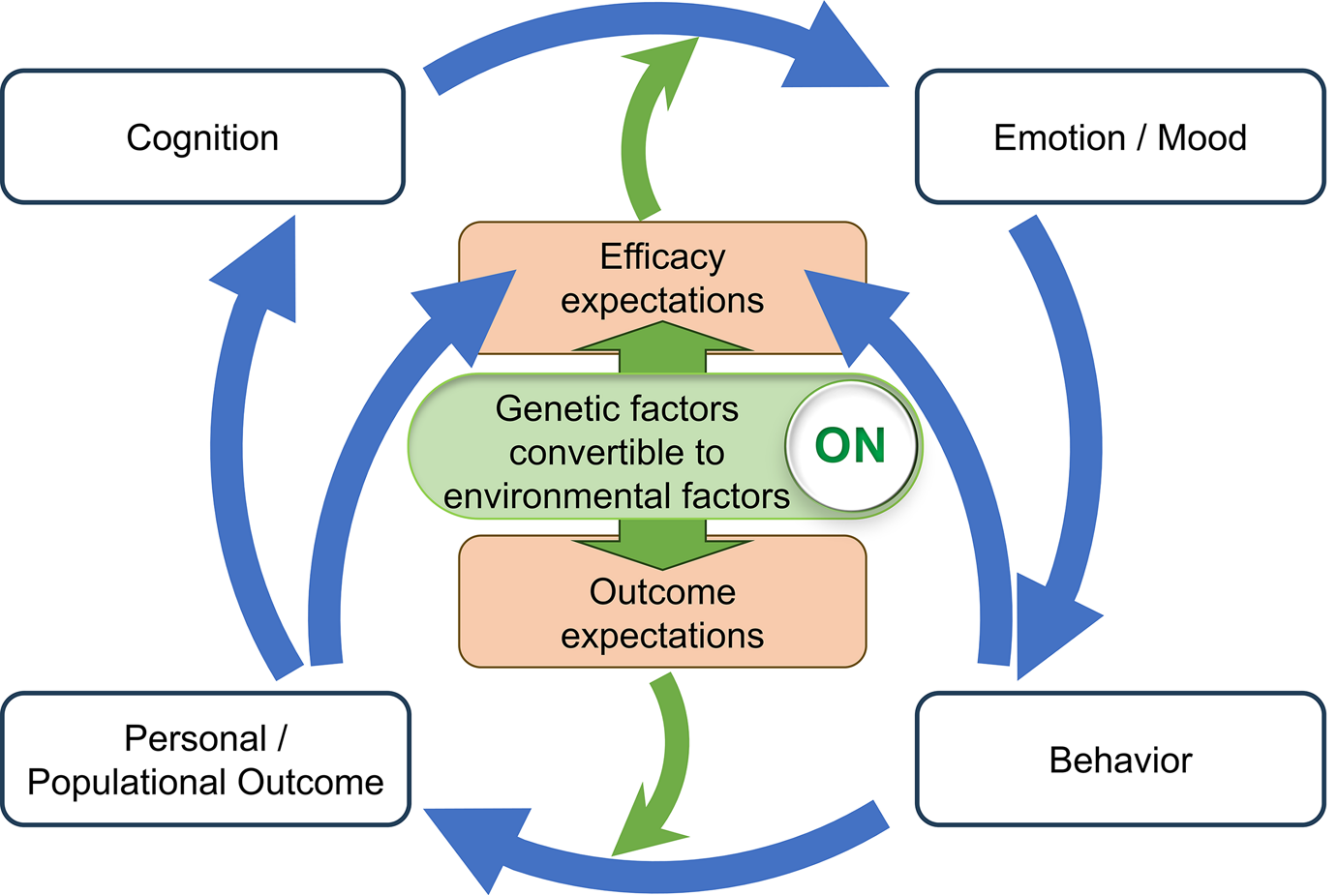
A theoretical framework for genome-personalized nursing on gout/hyperuricemia management. Based on a health-behavior intervention model from the behavioral change theory called social–cognitive theory (SCT), we mapped a theoretical framework for genome-personalized nursing on gout/hyperuricemia management onto the process. From the results of the present study, we concluded that the framework would function effectively with cognitive interventions by nurses through the convertibility of genetic factors into environmental factors, and could promote individuals’ appropriate health behavior based on the motivation of “efficacy expectations (self-efficacy)” and “outcome expectations” and positive feedback. This framework demonstrated the concept of “genome-personalized nursing” in the present study

Figure 1 shows the theoretical framework that we mapped onto the process, which is based on a psychological model from a behavioral change theory called SCT, formulated by Bandura [28]. When implementing genome-personalized nurse-led care, two expectations, i.e., efficacy expectations (self-efficacy) and outcome expectations, as to the framework (Fig. 1) have key problems due to both the “unchangeable” and “predictable” natures of individual genetic variants. Firstly, both characteristics are likely to reduce efficacy expectations and lead to negative emotions/mood, resulting in avoidant behavior. Secondly, they are likely to reduce outcome expectations, because individuals might doubt that a particular behavior based on their genetic variants will produce good outcomes on their disease.

The present study is the first to reveal the convertibility of genetic factors into environmental factors in terms of ability to increase SUA levels in both sexes. Individuals with mild dysfunction (3/4 function) of ABCG2, for example, could offset their genetic variants by reducing their BMI by 2.32 kg/m^2^ (males) and 1.71 kg/m^2^ (females) in a genome-personalized manner. These results on convertibility would be able to pull both triggers (Fig. 1) by improving the “predictable” onset of hyperuricemia/gout. Cognitive interventions by nurses on individual variants could therefore promote self-efficacy as well as outcome expectations, and produce positive emotions/mood that would lead to behavioral changes. Taken together, we concluded that the framework (Fig. 1) could function effectively with cognitive interventions by nurses through the convertibility of genetic factors into environmental factors, and could promote individuals’ appropriate health behavior and boost positive feedback.

## Discussion

The present study revealed the strong genetic effect on the progression of hyperuricemia from the viewpoint of population risk. The *ABCG2* variants had higher PAF for the progression of hyperuricemia than other typical environment risk factors, i.e., overweight/obesity, heavy drinking, and aging. By categorizing according to sex, the present study revealed that the PAF for aging carried no significant population risk in males, whereas heavy drinking showed no significance in females. All the factors showed a significant individual risk of increasing SUA level, but, unexpectedly, age displayed a slightly reverse association with SUA level in males. Overall, though, *ABCG2* variants were revealed to have sufficiently strong factors to exert both population and individual risks. In other words, genotyping *ABCG2* has likely benefits from the viewpoint of public health as well as of genome-personalized prevention/medicine. Notably, the convertibility of genetic factors into environmental factors would establish self-efficacy and outcome expectations based on the theoretical framework (Fig. 1) through cognitive interventions by nurses that would promote individuals’ appropriate health behaviors.

The results on high PAF for *ABCG2* indicated the importance of risk surveillance, or screening, from a public health viewpoint, because higher PAF means a higher frequency in the population as well as an elevated risk ratio. PAF for dysfunctional *ABCG2* variants reached approximately 30% in males, females, and in both populations, indicating that about 30% of hyperuricemia patients in the Japanese population originate from ABCG2 dysfunction. Together with an RR of approximately 1.8, these results for *ABCG2* variants were similar to those in our previous study that had 5005 Japanese participants [6]. A PAF of about 30% is too large to be ignored, especially as the PAF for smoking on cancer mortality is reportedly 29.8% in the Japanese male population [29]. The PAFs for overweight/obesity were about 15–20% in all and sex-stratified populations, which clearly demonstrated the importance of reducing body weight for lifestyle-related diseases in both the male and female populations. In contrast to the PAF for *ABCG2* variants and overweight/obesity, that for aging in males did not show any significance. Although aging is a typical risk factor for hyperuricemia, the present results might be explained by the birth cohort effect on SUA, which means that births during a specific period influence disease incidence; this is one of the limitations of cross-sectional studies. In fact, there is a cross-sectional study [30] which shows higher SUA levels in younger cohorts than in elder cohorts in a male population but not in females, resulting in a decline in SUA levels in males with increasing age. The PAF for heavy drinking showed no significance in females, which could be probably ascribed to the relatively low hyperuricemic population (*n* = 11) in 841 heavy-drinking females. Only 45 (1.01%) of 4466 females had hyperuricemia when the definition of hyperuricemia was set at an SUA of > 7 mg/dL, but this number increased to 211 (4.72%) when SUA was redefined as > 6.0 mg/dL. These results for PAF show that *ABCG2* variants and overweight/obesity at least were worth examining for hyperuricemia/gout prevention from a public health viewpoint.

Evaluation of effect size using regression coefficients revealed that all factors including genetic factors and typical environmental factors significantly and independently affected SUA; this was also consistent with our previous results [6]. As mentioned above, the reason that age in males was inversely proportional to SUA might be explained by the birth cohort effect. It is of interest that these results reveal the convertibility of genetic factors into environmental factors based on the ratios of regression coefficients (β_ABCG2_/β) and showed stronger effects of *ABCG2* variants in terms of ability to raise SUA. Males with a 25% loss of ABCG2 function, for example, show an effect equivalent to a 2.32-point increase in BMI, indicating that those with *ABCG2* variants could set a numerical goal for reducing their body weight (e.g., 6.7 kg for a 170 cm-tall male) to prevent hyperuricemia by offsetting their genetic background. In other words, the predictable effects caused by genetic variants could be reduced by setting specific numerical targets for environmental factors. Genome-personalized nursing for gout/hyperuricemia could therefore be promoted by recommendations to reduce body weight and alcohol intake to match individuals’ genetic variations, i.e., *ABCG2* variants.

From the above findings, *ABCG2* variants were revealed to have a larger PAF than other typical environmental factors, with their effect size being significant and comparatively greater than other lifestyle-related factors such as BMI and alcohol consumption. These findings indicate that *ABCG2* variants had sufficient effect size for them to be converted into other environmental factors and could thus play an important role in persuasion to adopt behavioral changes.

Based on this framework, two approaches might be used for health behavioral intervention: a population approach and a high-risk approach. To help prevent hyperuricemia/gout, the population approach would set as its goal a reduced population-wide SUA level, while the high-risk approach would aim to reduce SUA in individuals at high risk.

The high PAF identified in the present study reflects highly shared genetic variants in the Japanese population, indicating genetic screening to be potentially useful as health screening and/or intervention for obese individuals, heavy drinkers, and the elderly. It would also make it easy to perform genetic screening, since genotyping only two variants of *ABCG2* using a TaqMan assay, for example, is considerably less expensive than adopting next-generation sequencers. From the viewpoint of improving public health, it should be possible in future to use cost-effective genetic screening to reduce the burden of common diseases on healthcare systems. This would enable public health nurses to select a target population, i.e., those with the variants in question, for intervention. In the light of the high frequency of *ABCG2* variants in the Japanese population, health education could be performed on the impact of these genetic variants and the extent of the risk they represent as well as environmental factors. Practice nurses at hospitals then could provide health education matched to the genetic variants of hyperuricemia/gout patients to reduce SUA levels.

In addition to the Q126X and Q141K variants evaluated in the present study, there might be other genetic factors that affect hyperuricemia/gout with large effect sizes, such as rare variants of *ABCG2* [31] and variants of other genes [32–34]. For hyperuricemia/gout as a lifestyle disease, environmental factors as well as genetic factors cannot be ignored; it is important to provide explanations which do not mislead people into thinking that carrying no variants is equivalent to zero risk of hyperuricemia/gout. Moreover, there could be drawbacks to the implementation of genome-personalized nursing in other populations, due to variations in genetic factors such as minor allele frequencies of *ABCG2* variants and environmental factors, such as lifestyles. Further studies need to be performed that center on other populations.

In the present study, we revealed that dysfunctional variants of *ABCG2* carry a high individual and population risk of progression of hyperuricemia in both sexes. Based on the results for the convertibility of genetic factors into environmental factors, we also mapped the theoretical framework of a health behavior intervention model onto the process and examined whether the framework would function. We propose here for the first time the concept of “genome-personalized nursing” based on these findings for hyperuricemia and gout, i.e., as lifestyle-related diseases.

We conclude that a more appropriate high-risk approach for individuals and population approach can be supported under the present framework which takes advantage of the convertibility of genetic factors into environmental factors, and that the framework will not only be useful for T2T care, but also for a move toward genome-personalized nurse-led care.

## Supplementary Information

Below is the link to the electronic supplementary material.

Supplementary file1 (DOCX 353 KB)

## Data Availability

Data are available upon reasonable request to the corresponding author.
