# Supplementary material for: Dysfunctional variants of ABCG2 create strong individual and population risks for progression of hyperuricemia: the potential for implementation of genome-personalized nursing
Source: Hum Cell. 2025 Dec 26;39(1):22. doi: 10.1007/s13577-025-01310-y (PMC12743024; doi:10.1007/s13577-025-01310-y)
Supplement: Supplementary file 1 — Supplementary file1 (DOCX 353 KB) [file 13577_2025_1310_MOESM1_ESM.docx]

**Supplementary Information**

**Title:**

Dysfunctional variants of ABCG2 create strong individual and population risks for progression of hyperuricemia: the potential for implementation of genome-personalized nursing

**Journal name:**

*Human Cell*

**Authors:**

Akiyoshi Nakayama^1,2,*,†^, Kimiko Hayano^3,*^, Miki Ueno^4,*^, Yuka Miyoshi^5,6^, Hiroshi Nakashima^5^, Seiko Shimizu^1^, Itsumi Hashimoto^1^, Miho Toba^1^, Rion Kikuchi^1^, Nana Takehana^1^, Mitsuru Miura^1^, Yusuke Kawamura^1^, Yu Toyoda^1^, Tomoko Mizuno^1^, Risa Tanabe^7^, Yoshinobu Hamada^7^, Takashi Tamura^8^, Yasufumi Kato^8^, Yoko Mitsuda^8^, Hirofumi Nakaoka^9,10^, Ken Yamamoto^11^, Masashi Tsunoda^5^, Nariyoshi Shinomiya^1^, Hirotaka Matsuo^1,12,†^

**Affiliations:**

1. Department of Integrative Physiology and Bio-Nano Medicine, National Defense Medical College, 3-2 Namiki, Tokorozawa, Japan
2. International Research Collaboration Officer, National Defense Medical College Research Institute, National Defense Medical College, Tokorozawa, Japan
3. Department of Community Health Nursing, National Defense Medical College, Tokorozawa, Japan
4. Department of Nursing, National Defense Medical College, Tokorozawa, Japan
5. Department of Preventive Medicine and Public Health, National Defense Medical College, Tokorozawa, Japan
6. Research Division, Maritime Self-Defense Force Undersea Medical Center, Yokosuka, Japan
7. Department of Obstetrics and Gynecology, National Defense Medical college, Tokorozawa, Japan
8. Department of Preventive Medicine, Nagoya University Graduate School of Medicine, Nagoya, Japan
9. Department of Cancer Genome Research, Sasaki Institute, Sasaki Foundation, Tokyo, Japan
10. Department of Biomedical Data Science, Kagoshima University Graduate School of Medical and Dental Sciences, Kagoshima, Japan
11. Department of Medical Biochemistry, Kurume University School of Medicine, Kurume, Fukuoka, Japan
12. Department of Biomedical Information Management, National Defense Medical College Research Institute, National Defense Medical College, Tokorozawa, Japan

* These authors contributed equally to this work.

† These authors share senior authorship.

^†^**Correspondence authors:**

Hirotaka Matsuo, MD, PhD; Akiyoshi Nakayama, MD, PhD

Department of Integrative Physiology and Bio-Nano Medicine

National Defense Medical College, 3-2 Namiki, Tokorozawa, Saitama 359-8513, Japan

E-mail address: [matsuo29@gmail.com](mailto:matsuo29@gmail.com) (H.M.), [aknak@ndmc.ac.jp](mailto:aknak@ndmc.ac.jp) (A.N.)

**Contents:**

Supplementary Figure S1: PAF for hyperuricemia progression for each risk and each sex

Supplementary Figure S2: Effect of ABCG2 variants corresponds to other risk factors on serum uric acid (SUA) level

Supplementary Table S1: Characteristics of 9,244 Japanese participants

Supplementary Table S2: Conversion table of alcohol consumption

Supplementary Table S3: SUA in each ABCG2 functional group estimated from the combination of dysfunctional variants

Supplementary Table S4: PAF for hyperuricemia progression for each functional level of ABCG2

**
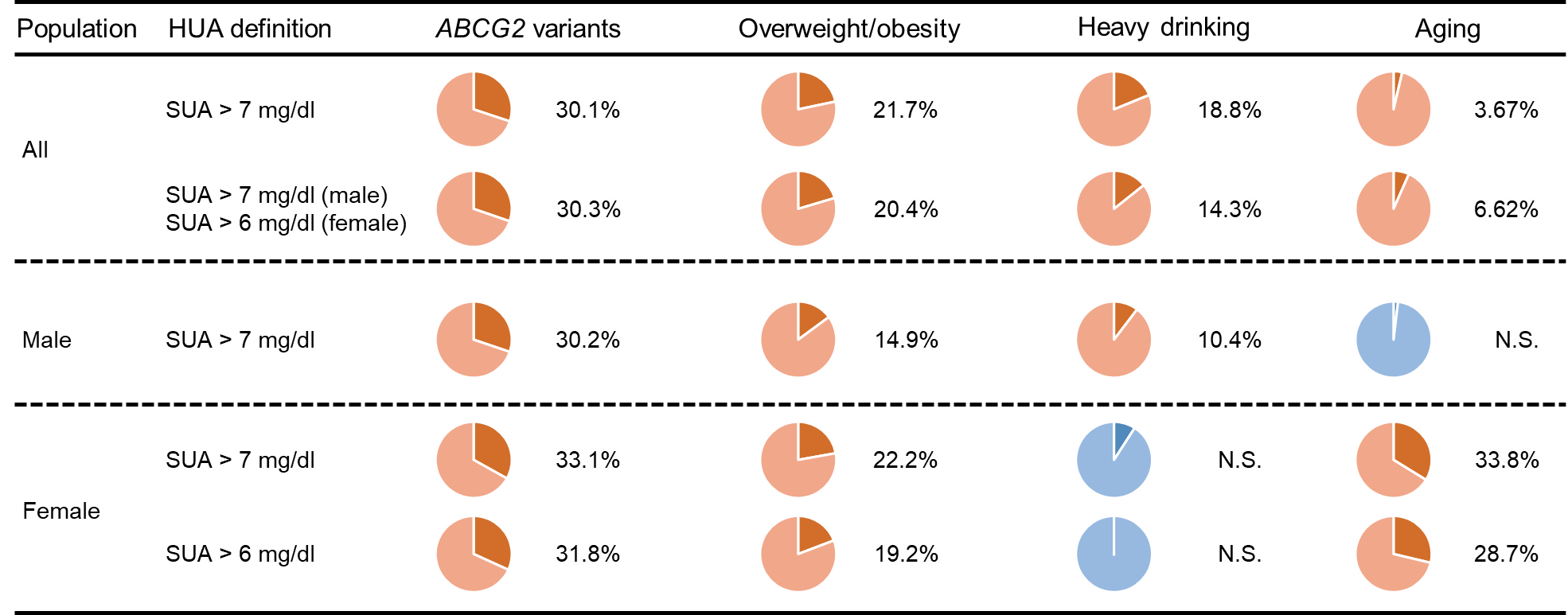
Supplementary Figure S1**: PAF for hyperuricemia progression for each risk and each sex

Abbreviations: HUA, hyperuricemia. SUA, serum uric acid level. N.S., not significant. PAF, population attributable fraction.


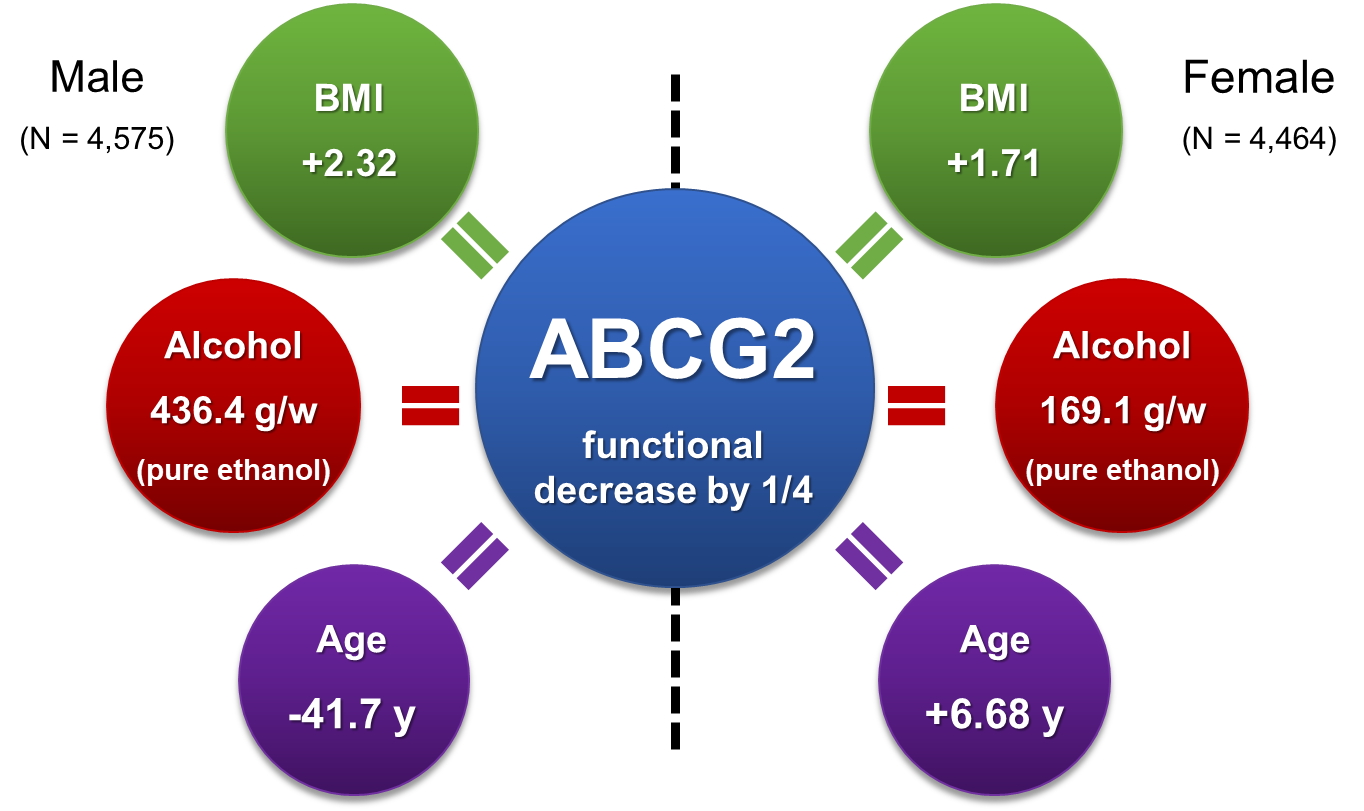


**Supplementary Figure S2**: Effect of ABCG2 variants corresponds to other risk factors on serum uric acid (SUA) level

The ratios of regression coefficients (β_ABCG2_/β) shown in Table 2 indicate the convertibility of genetic factor of *ABCG2* variants into environmental factors in terms of ability to SUA increase. These indicate that a decrease of 25% in ABCG2 function has the power to raise SUA levels comparable to ‘‘an increase of BMI by 2.32-point (= gaining a body weight of 6.7 kg for a 170 cm-tall male),’’ or ‘‘a consumption of pure ethanol by 436.4 g/w (= drinking 1.3 L of whiskey every week)” for male. For female, it can be then compared to “an increase of BMI by 1.71-point (= gaining body weight of 4.1 kg for a 155 cm-tall female),’’ or ‘‘consumption of pure ethanol by 169.1 g/w (= drinking 507 mL of whiskey every week).” These figures indicate that those with *ABCG2* variants could set a numerical goal for reducing their body weight or alcohol consumption to prevent hyperuricemia while offsetting genetic effects.

**Supplementary Table S1**: Characteristics of 9,244 Japanese participants

|  | Total |  | Shizuoka + Daiko areas | | |  | Shizuoka area | | |  | Daiko area | | |
| --- | --- | --- | --- | --- | --- | --- | --- | --- | --- | --- | --- | --- | --- |
|  |  |  | All male |  | All female |  | Male |  | Female |  | Male |  | Female |
| Number | 9,244 |  | 4,778 |  | 4,466 |  | 3,394 |  | 1,594 |  | 1,384 |  | 2,872 |
| SUA (mg/dl) | 5.24 ± 0.01 |  | 6.07 ± 0.02 |  | 4.36 ± 0.01 |  | 6.10 ± 0.02 |  | 4.44 ± 0.02 |  | 5.98 ± 0.03 |  | 4.32 ± 0.02 |
| Age (years) | 52.4 ± 0.10 |  | 53.0 ± 0.13 |  | 51.7 ± 0.15 |  | 52.8 ± 0.15 |  | 51.2 ± 0.22 |  | 53.6 ± 0.28 |  | 52.0 ± 0.19 |
| BMI (kg/m^2^) | 22.4 ± 0.03 |  | 23.4 ± 0.04 |  | 21.4 ± 0.05 |  | 23.5 ± 0.05 |  | 22.0 ± 0.08 |  | 23.2 ± 0.08 |  | 21.1 ± 0.06 |
| Alcohol consumption^a^  (g/week of pure alcohol) | 122.0 ± 2.08 |  | 184.0 ± 3.46 |  | 56.2 ± 1.72 |  | 157.5 ± 3.64 |  | 27.5 ± 1.79 |  | 248.6 ± 7.92 |  | 72.2 ± 2.44 |

Abbreviations: SUA, serum uric acid. BMI, body mass index.

^a^ See Supplementary Table S2 for the calculation of alcohol consumption.

Results are expressed as means ± S.E.

**Supplementary Table S2**: Conversion table for alcohol consumption

| Alcoholic drinks | Amount | Alcohol consumption  (grams of pure alcohol) |
| --- | --- | --- |
| Whiskey | a single shot (= 30 ml) | 10 |
|  | a double shot (= 60 ml) | 20 |
| Wine | a glass (=120 ml) | 12 |
| Beer | a large bottle (= 633 ml) | 25 |
|  | a mid-sized bottle (= 500 ml) | 20 |
|  | a 350-ml can or less | 14 |
|  | a 250-ml can or less | 7 |
| Japanese sake | 1 gou (=180 ml) | 22 |
| Shochu | 1 gou (=180 ml) | 50 |
| Shochu highball | 1 glass (= 350 ml) | 20 |

Referred from Ref. 6 in the main text under the terms of the Creative Commons Attribution-NonCommercial-NoDerivs 3.0 Unported License (https://creativecommons.org/licenses/by-nc-nd/3.0/).

**Supplementary Table S3**: SUA in each ABCG2 functional group estimated from the combination of dysfunctional variants

| Function of ABCG2 |  | Diplotype of Q126X and Q141K^a^ |  | Total | | |  | Male | | |  | Female | | |
| --- | --- | --- | --- | --- | --- | --- | --- | --- | --- | --- | --- | --- | --- | --- |
|  |  |  |  | *N_all_* (%)^b^ | *N* (%)^b^ | SUA |  | *N_all_* (%)^b^ | *N* (%)^b^ | SUA |  | *N_all_* (%)^b^ | *N* (%)^b^ | SUA |
| Full function |  | *1/*1 |  | 4,318  (46.7) | 4,274  (47.3) | 5.10 ± 0.02 |  | 2,240  (46.9) | 2,196  (48.0) | 5.90 ± 0.02 |  | 2,078  (46.5) | 2,078  (46.5) | 4.26 ± 0.02 |
| 3/4 function  (mild dysfunction) |  | *1/*2 |  | 3,664  (39.6) | 3,570  (39.5) | 5.29 ± 0.02 |  | 1,881  (39.4) | 1,788  (39.1) | 6.16 ± 0.03 |  | 1,783  (39.9) | 1,782  (39.9) | 4.41 ± 0.02 |
| 1/2 function  (moderate dysfunction) |  | *1/*3 or  *2/*2 |  | 1,143  (12.4) | 1,081  (12.0) | 5.38 ± 0.04 |  | 590  (12.3) | 529  (11.5) | 6.27 ± 0.06 |  | 553  (12.4) | 552  (12.4) | 4.53 ± 0.04 |
| ≤1/4 function  (severe dysfunction) |  | *2/*3 or  *3/*3 |  | 119  (1.3) | 114  (1.3) | 5.69 ± 0.13 |  | 67  (1.4) | 62  (1.4) | 6.40 ± 0.16 |  | 52  (1.2) | 52  (1.2) | 4.84 ± 0.15 |
| Total |  |  |  | 9,244  (100.0) | 9,039  (100.0) | 5.22 ± 0.01 |  | 4,778  (100.0) | 4,575  (100.0) | 6.05 ± 0.02 |  | 4,466  (100.0) | 4,464  (100.0) | 4.36 ± 0.01 |

Abbreviations: SUA, serum uric acid.

SUA is expressed as means ± SE (mg/dl).

^a^ *1, *2 and *3 represent haplotypes “C–C” (Q126 and Q141), “C-A” (Q126 and K141) and “T-C” (X126 and Q141) of two dysfunctional variants of *ABCG2* gene, p.Q126X (c.376C > T; rs72552713) and p.Q141K (c.421C > A; rs2231142), respectively.

^b^ “*N_all_*” represents the numbers of all 9,244 participants in the present study, while “*N*” indicates the numbers from 9,039 individuals who received no urate-lowering therapy against gout/hyperuricemia and had no past histories of gout among all 9,244 participants.

**Supplementary Table S4**: PAF for hyperuricemia progression for each functional level of ABCG2

| Population^a^ | Dysfunctional level of ABCG2^b^ |  | PAF (%) | 95% CI | Risk ratio | 95% CI | *P* value |
| --- | --- | --- | --- | --- | --- | --- | --- |
| All | Whole dysfunction  (any dysfunction) |  | 30.1 | 24.6 - 35.6 | 1.81 | 1.61 - 2.03 | 2.85 × 10^-24^ |
|  | 3/4 function  (mild dysfunction) |  | 19.3 | 14.9 - 23.8 | 1.70 | 1.50 - 1.92 | 3.46 × 10^-17^ |
|  | 1/2 function  (moderate dysfunction) |  | 9.83 | 7.44 - 12.4 | 2.14 | 1.83 - 2.50 | 1.24 × 10^-21^ |
|  | ≤ 1/4 function  (severe dysfunction) |  | 0.939 | 0.226 - 1.70 | 2.04 | 1.37 - 3.05 | 6.54 × 10^-4^ |
| All | Whole dysfunction  (any dysfunction) |  | 30.3 | 25.1 - 35.4 | 1.81 | 1.63 - 2.02 | 1.86 × 10^-28^ |
| (HUA: SUA > 6 mg/dl  in female) | 3/4 function  (mild dysfunction) |  | 19.3 | 15.1 - 23.4 | 1.70 | 1.51 - 1.91 | 7.86 × 10^-20^ |
|  | 1/2 function  (moderate dysfunction) |  | 9.88 | 7.70 - 12.1 | 2.15 | 1.86 - 2.48 | 3.26 × 10^-25^ |
|  | ≤1/4 function  (severe dysfunction) |  | 1.09 | 0.405 - 1.82 | 2.21 | 1.56 - 3.15 | 2.01 × 10^-5^ |
| Male | Whole dysfunction  (any dysfunction) |  | 30.2 | 24.9 - 35.3 | 1.81 | 1.62 - 2.03 | 9.72 × 10^-27^ |
|  | 3/4 function  (mild dysfunction) |  | 19.5 | 15.1 - 23.7 | 1.71 | 1.52 - 1.93 | 6.70 × 10^-19^ |
|  | 1/2 function  (moderate dysfunction) |  | 9.98 | 7.69 - 12.3 | 2.16 | 1.86 - 2.50 | 1.00 × 10^-23^ |
|  | ≤ 1/4 function  (severe dysfunction) |  | 0.753 | 0.0935 - 1.47 | 1.77 | 1.20 - 2.62 | 0.0721 |
| Female | Whole dysfunction  (any dysfunction) |  | 33.1 | 2.91 - 62.5 | 1.93 | 1.03 - 3.61 | 0.0372 |
|  | 3/4 function  (mild dysfunction) |  | 22.2 | -2.35 - 46.3 | 1.83 | 0.94 - 3.57 | 0.0710 |
|  | 1/2 function  (moderate dysfunction) |  | 7.28 | -4.27 - 20.2 | 1.88 | 0.76 - 4.63 | 0.164 |
|  | ≤ 1/4 function  (severe dysfunction) |  | 3.67 | -0.957 - 10.6 | 5.71 | 1.33 - 24.5 | 8.87 × 10^-3^ |
| Female | Whole dysfunction  (any dysfunction) |  | 31.8 | 18.5 - 45.0 | 1.87 | 1.41 - 2.48 | 1.04 × 10^-5^ |
| (HUA: SUA > 6 mg/dl  in female) | 3/4 function  (mild dysfunction) |  | 19.7 | 8.86 - 30.5 | 1.72 | 1.27 - 2.33 | 3.77 × 10^-4^ |
|  | 1/2 function  (moderate dysfunction) |  | 9.56 | 3.89 - 15.5 | 2.13 | 1.45 - 3.14 | 9.85 × 10^-5^ |
|  | ≤ 1/4 function  (severe dysfunction) |  | 2.52 | 0.335 - 5.07 | 4.18 | 2.02 - 8.65 | 6.84 × 10^-5^ |

Abbreviations: PAF, population attributable fraction. CI, confidence interval. HUA, hyperuricemia. SUA, serum uric acid level.

^a^ Hyperuricemia was defined for both sexes when their serum uric acid (SUA) level was over 7 mg/dl. It was set as over 6 mg/d for female if indicated.

^b^ “Whole dysfunction (any dysfunction)” indicates the total value of “3/4 dysfunction,” “1/2 dysfunction” and “≤1/4 dysfunction.”
